# *Leucaena leucocephala* leachate compromised membrane integrity, respiration and antioxidative defence of water hyacinth leaf tissues

**DOI:** 10.1186/1999-3110-54-8

**Published:** 2013-08-21

**Authors:** Tsun-Thai Chai, Keng-Fei Ooh, Pei-Wan Ooi, Pei-Sing Chue, Fai-Chu Wong

**Affiliations:** 1 Department of Chemical Science, Faculty of Science, Universiti Tunku Abdul Rahman, Kampar, 31900 Malaysia; 2 Centre for Biodiversity Research, Universiti Tunku Abdul Rahman, Kampar, 31900 Malaysia

**Keywords:** Allelopathy, Ascorbate peroxidase, Catalase, *Eichhornia crassipes*, Electrolyte leakage, *Leucaena leucocephala*, Reactive oxygen species, Respiration

## Abstract

**Background:**

Water hyacinth is an invasive aquatic weed in many regions of the world. In this study, the bioherbicidal potential of allelopathic plant *Leucaena leucocephala* against water hyacinth was investigated using a leaf disc assay.

**Results:**

*L. leucocephala* leachate enhanced electrolyte leakage from water hyacinth leaf discs in a concentration-dependent manner. Control experiments eliminated the possibilities that increased membrane permeability in the leachate-treated leaf discs was due to pH or osmotic effects of the leachate. Thus, the loss of membrane stability in the leachate-treated leaf discs was likely due to phytotoxins detected in the leachate, namely mimosine and phenolic constituents. Decline in tissue respiration was detected in leachate-treated water hyacinth leaf discs. This suggests that the *L. leucocephala* leachate may contain compounds which acted as respiratory inhibitors. Enhanced reactive oxygen species production coincided with inhibition of catalase and ascorbate peroxidase activities in the leachate-treated water hyacinth leaf tissues. The injurious effects of *L. leucocephala* leachate on water hyacinth leaf discs probably involved direct inhibition of antioxidant enzymes in addition to direct involvement of some allelochemicals in reactive oxygen species formation.

**Conclusion:**

In summary, the toxic effects of *L. leucocephala* leachate on water hyacinth leaf discs likely lay in its ability to effectively compromise the membrane integrity, tissue respiration and antioxidant defence of the latter.

**Electronic supplementary material:**

The online version of this article (doi:10.1186/1999-3110-54-8) contains supplementary material, which is available to authorized users.

## Background

Water hyacinth, *Eichhornia crassipes* (Mart.) Solms., is a floating aquatic plant native to Brazil. This species is an invasive weed in numerous freshwater ecosystems between 40°N and 40°S worldwide. Water hyacinth is fast-growing and often forms dense mats on lakes, rivers, and waterways. Rapid, uncontrolled proliferation of water hyacinth adversely affects aquatic fauna and flora, impacting local biodiversity seriously. Colonisation of water bodies by water hyacinth poses problems to human activities, such as agriculture, recreation, power generation and transportation (Malik, 2007).

The application of allelochemicals, in either pure or crude form, is a potentially valuable and sustainable approach in aquatic weed control (Szczepanski, 1977; Singh et al., 2003). Being natural plant products, allelochemicals are considered relatively eco-friendly as they are likely to degrade rapidly in the environment. The effectiveness of powders or extracts of allelopathic plants in inhibiting the growth of water hyacinth or killing the weed has been demonstrated (Pandey et al., 1993; Kathiresan, RM RM 2000; Saxena, 2000).

*Leucaena leucocephala* (Fabaceae) is an allelopathic tree species that is widespread in tropics and subtropics. Phytotoxic allelochemicals identified in the leaves of this legume species include mimosine and phenolic compounds, such as quercetin, p-hydroxycinnamic acid, protocatechuic acid, and gallic acid (Chou and Kuo, 1986). The physiological mechanisms of allelochemicals are complicated, and the outcome of an allelopathic interaction between two plants is often species-dependent (Iftikhar Hussain et al., 2008). Although the bioherbicidal activity of *L. leucocephala* on other terrestrial plants is well-established (Hong et al., 2003; John and Narwal, 2003), little is known about its effects on water hyacinth or other aquatic weeds.

Phenolic allelochemicals often adversely affect the membrane stability, respiration and oxidative status of plant cells (Einhellig, 2004; Weir et al., 2004; Gniazdowska and Bogatek, 2005; Li et al., 2010). Mimosine is known to inhibit the activities of iron-containing enzymes, some of which are important antioxidant enzymes in plant cells, e.g. catalase and peroxidase (Prasad and Subhashini, 1994; Andrade et al., 2008). Hence, there are two objectives in this study: first, to confirm whether *L. leucocephala* leachate is phytotoxic to water hyacinth, based on its effects on membrane integrity and tissue respiration; second, to confirm whether any phytotoxicity detected is mediated by suppression of antioxidative defence.

## Methods

### Preparation of *Leucaena leucocephala* leachates

Healthy mature leaves were collected from *Leucaena leucocephala* trees grown on the Kampar campus of Universiti Tunku Abdul Rahman. Leaflets were removed from the petioles, briefly washed and blotted dry. The leachates (5%) used in this study were prepared by soaking 5 g of leaf material in 100 mL of autoclaved deionised water for 24 h and 48 h, respectively. Incubation of the leaf material was carried out at 25°C in darkness on an orbital shaker (90 rpm). Using vacuum-filtration, leaf material was removed from the leachate solutions. Remaining particles were then removed by centrifugation at 8603 ×g and 4°C for 10 min. The supernatants obtained were stored at −20°C until used.

### Experiment 1A: Effects of leachates on cellular membrane injury and tissue respiration

Healthy water hyacinth plants with similar sizes were collected from a lake next to the campus at 9 am in the morning. The plants were carefully rinsed under running tap water to remove any debris, silt or small invertebrates that were present. The plants were then placed in a large plastic box filled with distilled water and allowed to acclimate for 24 h (12 h/12 h light/dark) at room temperature. The plants were illuminated at 100 μmol photons/m^2^/s Photosynthetically Active Radiation (PAR) during the light period. The next day, leaf discs (0.7 cm diameter) were cut from uniform-looking leaves with a cork borer and then rinsed twice with autoclaved deionised water. Next, the leaf discs were vacuum-infiltrated (five cycles of 20 s) with different dilutions of leachates (0, 0.1, 0.5, 1.0, 2.5, and 5.0%) and then transferred to Petri dishes each containing 20 mL of the same leachate solution, supplemented with 50 μL of Tween-20. The Petri dishes were shaken on an orbital shaker (90 rpm) for 24 h at 25°C in darkness. At the end of the treatment, the leaf discs were rinsed with deionised water, blotted dry and used for analyses. Cellular membrane injury was assessed by measuring relative electrolyte leakage (REL) of the leaf discs as previously described (Kraus and Fletcher, 1994). Tissue respiration of the leaf discs was determined indirectly using 2,3,5-triphenyl tetrazolium chloride (TTC) as described in (Steponkus and Lanphear 1967). Reduction of TTC was expressed as absorbance per g dry weight (DW).

### Experiment 1B: Effects of leachate pH and osmotic potential on cellular membrane injury

The pH and osmotic potential of *L. leucocephala* leachates (0.1, 0.5, 1.0, 2.5 and 5.0%) were determined and the effects of these two parameters on water hyacinth leaf discs were evaluated. The pH values of different dilutions of 24 h and 48 h leachates ranged between 6.9 and 7.5. To determine whether the pH of the leachates contributed to cellular membrane injury, leaf discs were treated as described above for Experiment 1A. Briefly, leaf discs were vacuum-infiltrated with deionised water pre-adjusted to pH 6.5, 7.0, 7.5, and 8.0 with 0.1 M NaOH or 0.1 M HCl. The leaf discs were then incubated in the same pH solutions for 24 h. REL of the treated leaf discs was determined according to (Kraus and Fletcher 1994).

The osmotic potential of 24 h and 48 h leachates was estimated based on electrical conductivity (EC) measurements and the relation of osmotic potential (MPa) = −0.036 × EC, with EC in dS/m (Bingham et al., 1987; Raviv and Blom, 2001). Electrical conductivity of the leachates was measured using a conductivity meter (Oakton Instruments, Vernon Hills, Illinois, USA). The osmotic potential of different dilutions of 24 h and 48 h leachates (0.1, 0.5, 1.0, 2.5 and 5.0%) ranged between −0.001 MPa and −0.036 MPa as well as between −0.002 and −0.079 MPa, respectively. To investigate possible effects of leachate osmotic potential on cellular membrane injury, leaf discs were treated as described above for Experiment 1A with mannitol solutions adjusted to the following osmotic potentials: -0.05, -0.10, and −0.15 MPa. Mannitol concentrations corresponding to the aforementioned osmotic potential values were 2.532, 5.064, and 7.597 g/L, respectively, calculated from data published by (Sosa et al. 2005). Deionised water (osmotic potential taken as 0.0 MPa) was used as control. REL of the leaf discs was determined as previously described (Kraus and Fletcher, 1994).

### Experiment 2: Detection of reactive oxygen species (ROS) production

Water hyacinth leaf discs were treated with 48 h leachate at different concentrations (0, 1.0, 2.5 and 5.0%) as described above for Experiment 1A. At the end of the 24 h treatment period, the leaf discs were used for detection of ROS accumulation. Hydrogen peroxide (H_2_O_2_) content of the leaf tissues was determined as described in (Velikova et al. 2000) and expressed as nmol/g fresh weight (FW). Visualisation of superoxide (O_2_^●-^) production in 0, 2.5, and 5% leachate-treated leaf discs was carried out as described in (Dutilleul et al. 2003).

### Experiment 3: Assays of catalase (CAT) and ascorbate peroxidase (APX) activities

Water hyacinth leaf discs were treated with 48 h leachate at different concentrations (0, 1.0, 2.5 and 5.0%) as described above for Experiment 1A. CAT specific activity of the leachate-treated leaf tissues was determined as described in (Dhindsa et al. 1981) and expressed as μmol H_2_O_2_ consumed/min/mg protein. APX specific activity of the leachate-treated water hyacinth leaf tissues was assayed according to (Nakano and Asada 1981) and expressed as nmol ascorbate oxidised/min/mg protein. Protein concentration of enzyme extracts was determined according to (Bradford 1976) using bovine serum albumin as the standard.

### Experiment 4: Phytochemical analysis

Total polyphenol and flavonoid contents of 24 h and 48 h leachates (5%) were determined as previously described (Chai and Wong, 2012). Polyphenol contents were expressed in mg gallic acid equivalents (GAE)/mL, whereas flavonoid content was expressed in mg quercetin equivalents (QE)/mL. Total contents of hydroxycinnamic acid derivatives were determined as in (Matkowski et al. 2008) and expressed in mg caffeic acid equivalents (CAE)/mL. Mimosine contents of the leachates were determined by HPLC using a Gemini 5u C18 110A column (4.6 mm × 250 mm, Phenomenex, Torrance, CA, USA). Mimosine was eluted with 0.2% orthophosphoric acid (v/v) in deionised water at a flow rate of 0.25 μL/min and was detected at a wavelength of 280 nm (Soedarjo and Borthakur, 1996). In our system, mimosine had a retention time of 6.3 min.

### Statistical analysis

Data reported are means ± standard errors. Statistical analysis was performed using SAS (version 9.2). Data were analysed by the ANOVA test and means of significant differences were compared using Student’s T-test at the 0.05 level of probability.

## Results

**Figure 1:**
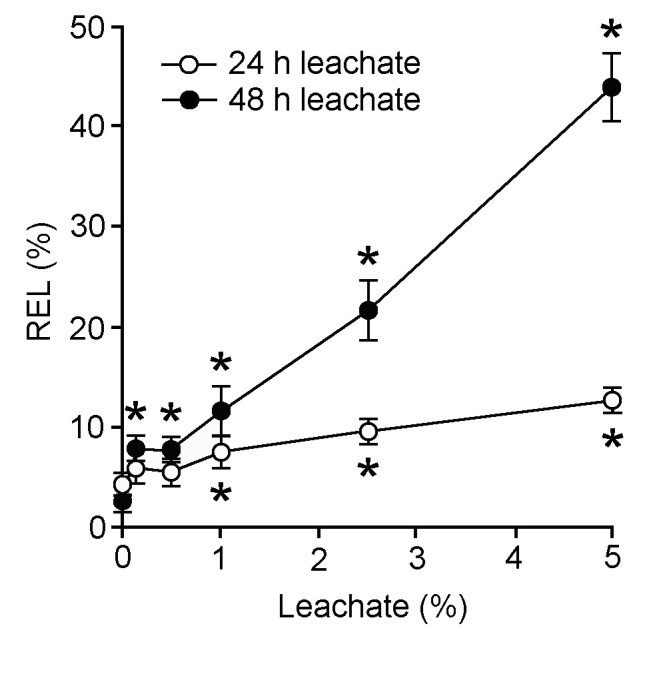
**Effects of different concentrations of 24 h and 48 h leachates on relative electrolyte leakage (REL) of water hyacinth leaf discs.** Data are presented as mean ± standard errors (*n* = 4). Asterisk (*) indicates significant difference from the control (0% leachate) as determined by Student’s T-test at *p* < 0.05.

Cellular membrane injury was evaluated in the *L. leucocephala* leachate-treated water hyacinth leaf discs based on REL measurements (Figure 1). Both of the 24 h and 48 h leachates caused cellular membrane injury, correlated with leachate concentrations. Notably, the 48 h leachate induced a more drastic increase in REL compared with 24 h leachate.

Water hyacinth leaf discs were treated with pH-adjusted water (pH 6.5, 7.0, 7.5 and 8.0) and mannitol solutions (0.00, -0.05, -0.10, and −0.15 MPa) to assess possible effects of leachate pH and osmotic potential to REL detected in leachate-treated leaf discs. REL of leaf discs treated with pH-adjusted water ranged between 4.9% and 5.3% (data not shown). REL of leaf discs treated with mannitol solutions ranged between 4.5% and 5.1% (data not shown). Neither pH nor osmotic treatments induced any significant changes in the REL of leaf discs.

**Figure 2:**
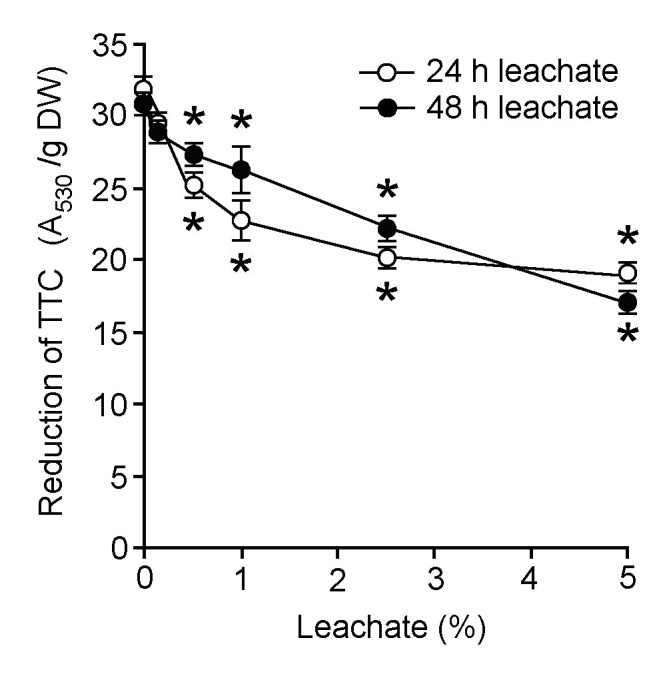
**Effects of different concentrations of 24 h and 48 h leachates on reduction of 2,3,5-triphenyl tetrazolium chloride (TTC) by water hyacinth leaf discs.** Data are presented as mean ± standard errors (*n* = 4). Asterisk (*) indicates significant difference from the control (0% leachate) as determined by Student’s T-test at *p* < 0.05.

Tissue respiration in leachate-treated water hyacinth leaf discs was assessed by measuring the reduction of TTC (Figure 2). Overall, 24 h and 48 h leachates both decreased the levels of TTC reduction by the leaf discs in a leachate concentration-dependent manner. The degree of decline in TTC reduction was generally similar in leaf discs treated with 24 h and 48 h leachates. At 5%, both types of leachates decreased TTC reduction to about 40% below the control level.

**Figure 3:**
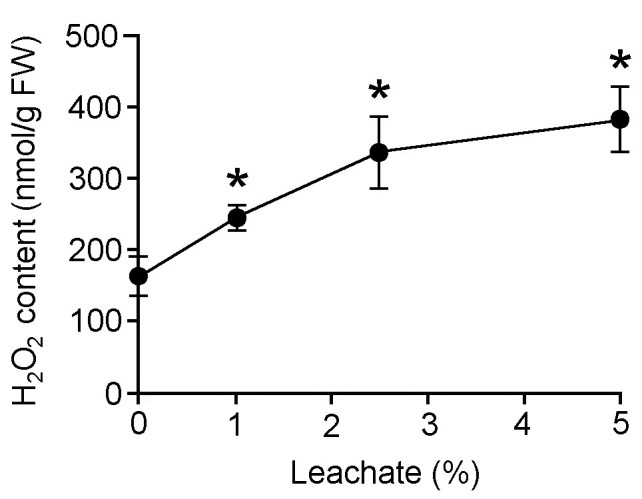
**Effects of different concentrations of 48 h leachate on hydrogen peroxide (H**_**2**_**O**_**2**_**) content of water hyacinth leaf discs.** Data are presented as mean ± standard errors (*n* = 4). Asterisk (*) indicates significant difference from the control (0% leachate) as determined by Student’s T-test at *p* < 0.05.

**Figure 4:**
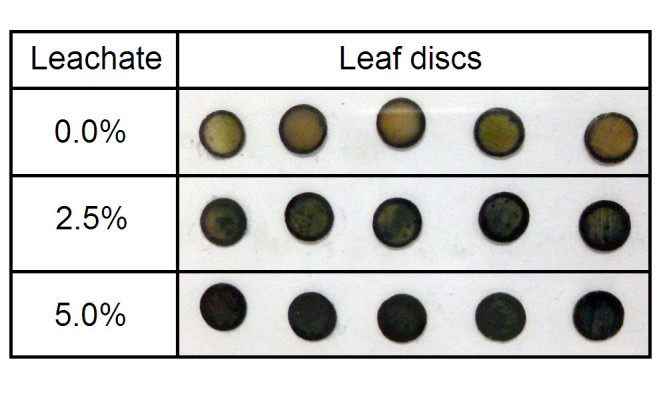
***In situ*** **tissue localisation of superoxide generation in water hyacinth leaf discs treated with 48 h leachate at different concentrations.** Formation of dark blue spots all over the leaf discs treated with 2.5 and 5% leachates indicate greater levels of superoxide production compared with control leaf discs treated with deionised water (0% leachate).

Treatment with 48 h leachate clearly increased ROS production in water hyacinth leaf discs. H_2_O_2_ content in the leaf discs increased in a leachate concentration-dependent manner (Figure 3). At 1, 2.5, and 5% leachate concentrations, H_2_O_2_ content in the leaf tissues increased to 1.5-, 2.1- and 2.4-fold higher than the control level. In addition, enhanced production of O_2_^●-^ was also detected in leaf discs treated with 2.5 and 5% 48 h leachates (Figure 4).

**Figure 5:**
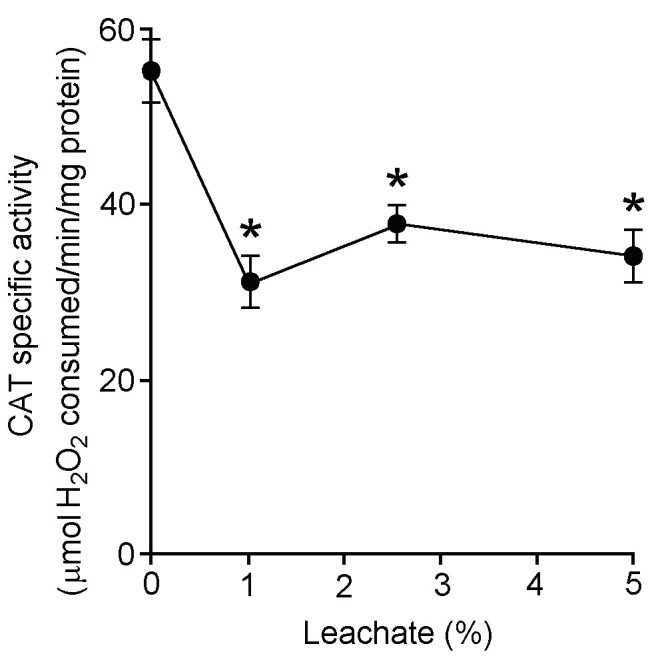
**Effects of different concentrations of 48 h leachate on catalase (CAT) specific activity of water hyacinth leaf tissues.** Data are presented as mean ± standard errors (*n* = 4). Asterisk (*) indicates significant difference from the control (0% leachate) as determined by Student’s T-test at *p* < 0.05.

**Figure 6:**
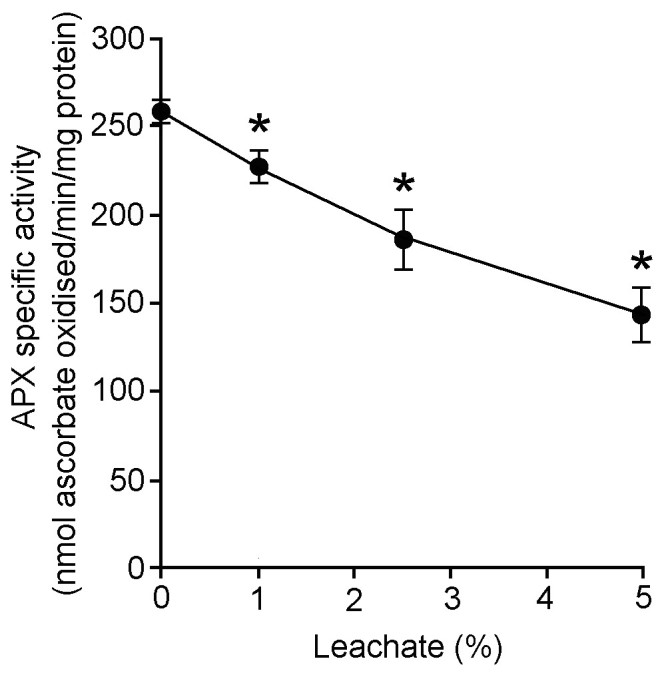
**Effects of different concentrations of 48 h leachate on ascorbate peroxidase (APX) specific activity of water hyacinth leaf tissues.** Data are presented as mean ± standard errors (*n* = 4). Asterisk (*) indicates significant difference from the control (0% leachate) as determined by Student’s T-test at *p* < 0.05.

CAT specific activities were repressed in water hyacinth leaf tissues treated with 48 h leachate (Figure 5). At 1, 2.5, and 5% leachate concentrations, CAT specific activities were inhibited by 43, 33, and 37% when compared with the control. Likewise, APX specific activities were inhibited in water hyacinth leaf tissues treated with 48 h leachate (Figure 6). In leaf discs treated with 1, 2.5, and 5% leachates, APX specific activities were progressively reduced by 11, 28, and 43% when compared with the control.

Phytochemical analysis found that the phenolic contents of 48 h leachate were overall about 2.8-fold higher compared with 24 h leachate (Table 1). Mimosine content in the 48 h leachate was 3.3-fold higher compared with the 24 h leachate. Correlation analysis between phytochemical contents of *L. leucocephala* leachates and REL values of leachate-treated leaf discs revealed high R^2^ values (R^2^ = 0.94, *p* < 0.05) (Table 2). By contrast, moderate R^2^ values (R^2^ = 0.51–0.55, *p* < 0.05) were obtained between phytochemical contents of leachates and TTC reduction of leachate-treated leaf discs.

**Table 1:** **Phytochemical contents of 5% leachate of** ***L. leucocephala*** **leaves**

Leachate	Total polyphenols (mg GAE/mL)	Total flavonoids (mg QE/mL)	Total hydroxycinnamic acids (mg CAE/mL)	Mimosine (mg/mL)
24 h	0.49 ± 0.01	0.52 ± 0.01	0.13 ± 0.01	0.28 ± 0.00
48 h	1.31 ± 0.02	1.45 ± 0.03	0.36 ± 0.01	0.91 ± 0.04

Data are means ± SE (*n* = 3). The values for 48 h leachate were all significantly different from those of the 24 h leachate according to Student’s T test at *p* < 0.05.

**Table 2:** **Correlation between phytochemical contents of** ***L. leucocephala*** **leachates and REL and TTC reduction of leachate-treated water hyacinth leaf tissues**

	Correlation of determination (R^2^)		
Parameters	Total flavonoids	Total hydroxycinnamic acids	Mimosine
REL	0.943	0.943	0.942
TTC reduction	0.507	0.554	0.512

R^2^ values presented were all statistically significant (*p* < 0.05).

## Discussion

*Leucaena leucocephala* is a well-studied allelopathic plant (John and Narwal, 2003). However, little is known about the phytotoxicity of *L. leucocephala* against aquatic weeds. In this study, we investigated effects of *L. leucocephala* leachate against water hyacinth leaf. Our findings demonstrated the potential of *L. leucocephala* as a bioherbicidal agent against water hyacinth.

Our REL results indicate that plasma membrane functions of water hyacinth leaf cells were disrupted by leachates of fresh leaves of *L. leucocephala*. REL assay is a sensitive test for the identification of allelochemicals, herbicides, or other compounds that destabilise cellular membranes (Dayan et al., 2000; Hoagland and Williams, 2004). Enhanced REL is also an indicator of cell death induction (Vidal et al., 2007; Luo et al., 2010; Mur et al., 2012). Hence, our findings clearly demonstrated the deleterious effects of *L. leucocephala* leachate on water hyacinth leaf tissues.

The loss of membrane stability in the leachate-treated leaf discs can be attributed to the phytotoxic chemical constituents in the leachates. Several lines of evidence support this proposal. Firstly, our control experiments eliminated the possibilities that the increased membrane permeability in the leachate-treated leaf discs was due to pH or osmotic effects of the leachates. Secondly, the higher phenolic and mimosine contents in the 48 h leachate relative to 24 h leachate paralleled the greater effects of the 48 h leachate on REL. Thirdly, our correlative analysis revealed that more than 90% of variation in REL may be accounted for by changes in the phytochemical contents measured. Finally, our findings are consistent with earlier reports of hydroxycinnamic acids, flavonoids and mimosine being phytotoxins in *L. leucocephala*(Chou and Kuo, 1986; John and Narwal, 2003; Chou, 2010).

In addition to loss of membrane integrity, decline in mitochondrial respiration activities was observed in the leachate-treated leaf tissues. Quercetin and 18 other flavonoids have been identified in the leaves of *L. leucocephala* (John and Narwal, 2003). Allelopathic flavonoids are known to act as inhibitors of the electron transport chain in the inner mitochondrial membrane (Einhellig, 2004). Mimosine, on the other hand, can cause mitochondrial dysfunction through rapid depolarisation of mitochondrial membranes in animal cells (Hallak et al., 2008). Hence, compromised dark respiration in the leachate-treated leaf discs may be attributed to the presence of flavonoids and/or mimosine which we detected in the leachate. Tissue respiration of leachate-treated leaf discs, nevertheless, correlated only moderately with the phenolic and mimosine contents of the leachate. This implies that respiratory inhibition may not be the primary mechanism in the phytotoxicity of *L. leucocephala* leachate.

Maintenance of plant membrane functions is an energy-demanding process. However, the lack of close correspondence between our REL and respiration data implies that disruption in energy metabolism alone could not fully explain the perturbations of plasma membrane functions in the leachate-treated leaf discs. ROS, through their peroxidative action on membrane lipids, were likely a key contributor to the loss of membrane integrity in the leachate-treated water hyacinth leaf discs. Some effects on the leaf cell membranes could also be due to the direct actions of phenolic constituents in the leachate. Cinnamic and benzoic acid derivatives, for example, can directly alter membrane proteins, resulting in structural changes in plasma membranes and consequently an increase in non-specific ion efflux (Einhellig, 2004).

In this study, enhanced ROS production coincided with repression of CAT and APX activities in the leachate-treated water hyacinth leaf tissues. Our findings suggest that the *L. leucocephala* leachate may have rendered the water hyacinth leaf tissues vulnerable to oxidative injury by direct inhibition of antioxidant enzymes. This is plausible considering that mimosine, which we detected in the leachate, can inhibit catalase and peroxidases in leaf and root tissues (Prasad and Subhashini, 1994; Andrade et al., 2008). Furthermore, phenolic compounds such as gallic and caffeic acids can directly induce cellular ROS formation (Isuzugawa et al., 2001; Singh et al., 2009). Hence, the potential role of mimosine in inducing oxidative stress in the leachate-treated leaf discs was possibly exacerbated by phenolic allelochemicals in the leachate.

The practical application of using *L. leucocephala* to control water hyacinth requires further investigations. As *L. leucocephala* is a terrestrial species, its use to control water hyacinth may entail foliar spraying of leachates or extracts of *L. leucocephala* over a water hyacinth colony. Foliar spraying of *Lantana camara* leaf extract has been shown to suppress leaf bud emergence in water hyacinth and induce leaf tissue decay (Zheng et al., 2006). Hence, foliar spraying of *L. leucocephala* leachates or extracts may be an effective means to control water hyacinth plants in the field, although this must be confirmed in future experiments. The addition of dried powder of *Coleus amboinicus* leaves into water has been demonstrated to cause the death of water hyacinth plants (Kathiresan, RM 2000). Thus, an alternative to foliar spraying is by adding *L. leucocephala* leaf material directly into a water hyacinth-infested water body and allowing it to decompose. In any case, the potential effects of *L. leucocephala* leachate, extract or leaf material on other organisms in the water body should be considered when assessing the feasibility of using *L. leucocephala* as a bioherbicidal agent to control water hyacinth.

## Conclusion

In conclusion, *L. leucocephala* leachate effectively attenuated plasma membrane functions, tissue respiration and antioxidant defence of the water hyacinth leaf tissues. Analyses carried out suggest that multiple phytotoxic compounds in the leachate may be acting in concert to compromise multiple target sites at the cellular level. More detailed biochemical and molecular studies are desired to further elucidate the modes of action of the leachate on specific target sites. Our study has demonstrated the bioherbicidal potential of *L. leucocephala* leachate on water hyacinth leaf discs under *in vitro* conditions. Future investigations using a whole-plant assay are required to confirm if *L. leucocephala* leachate has similar effects on intact water hyacinth plants under more natural conditions.

## Authors’ original submitted files for images

Below are the links to the authors’ original submitted files for images. Authors’ original file for figure 1 Authors’ original file for figure 2 Authors’ original file for figure 3 Authors’ original file for figure 4 Authors’ original file for figure 5 Authors’ original file for figure 6
