# Supplementary figures and images for: Leucaena leucocephala leachate compromised membrane integrity, respiration and antioxidative defence of water hyacinth leaf tissues
Source: Bot Stud. 2013 Aug 21;54:8. doi: 10.1186/1999-3110-54-8 (PMC5430313; doi:10.1186/1999-3110-54-8)

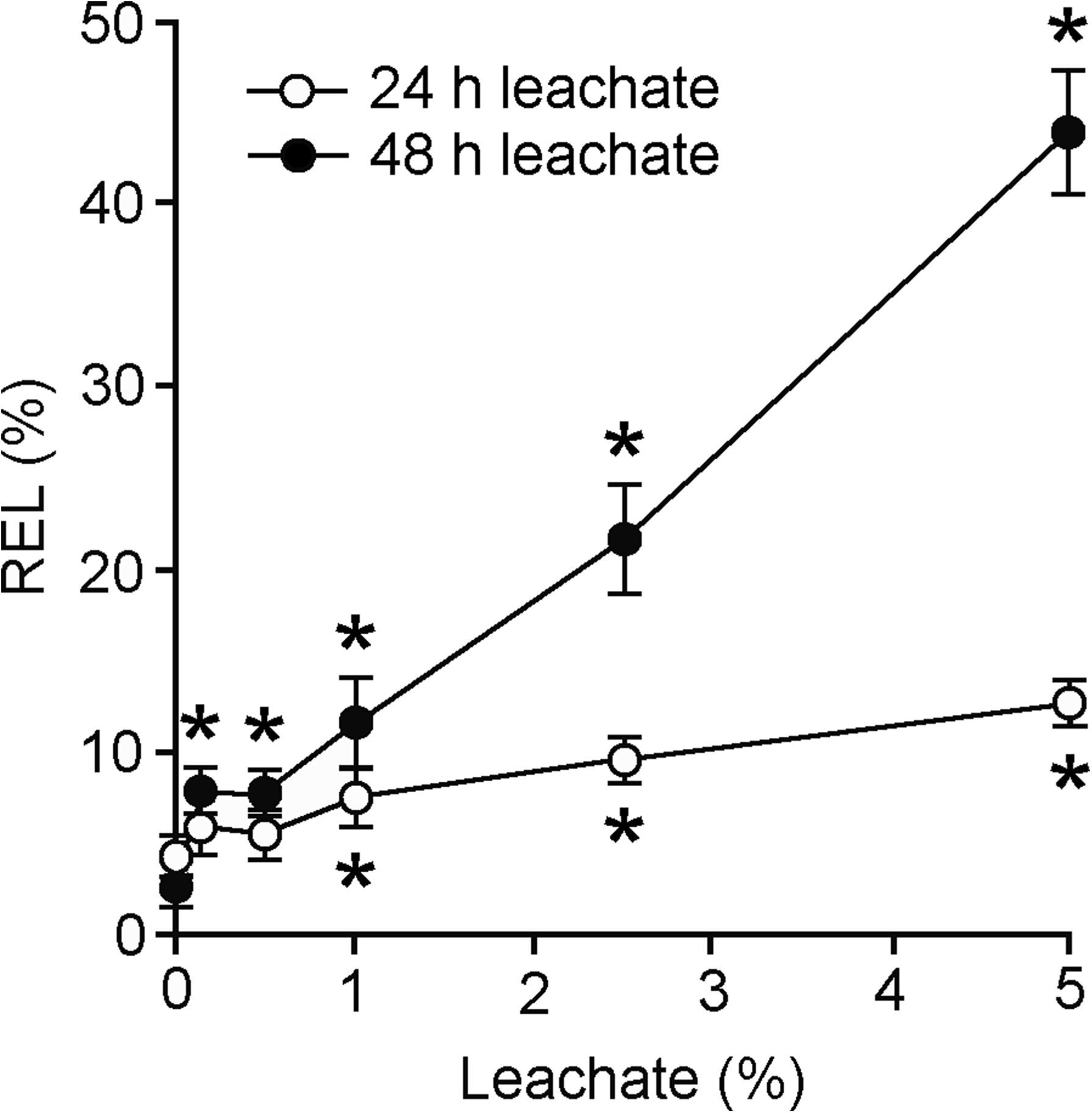

Supplement: Supplementary file 1 — Authors’ original file for figure 1 [file 40529_2012_10_MOESM1_ESM.tif]

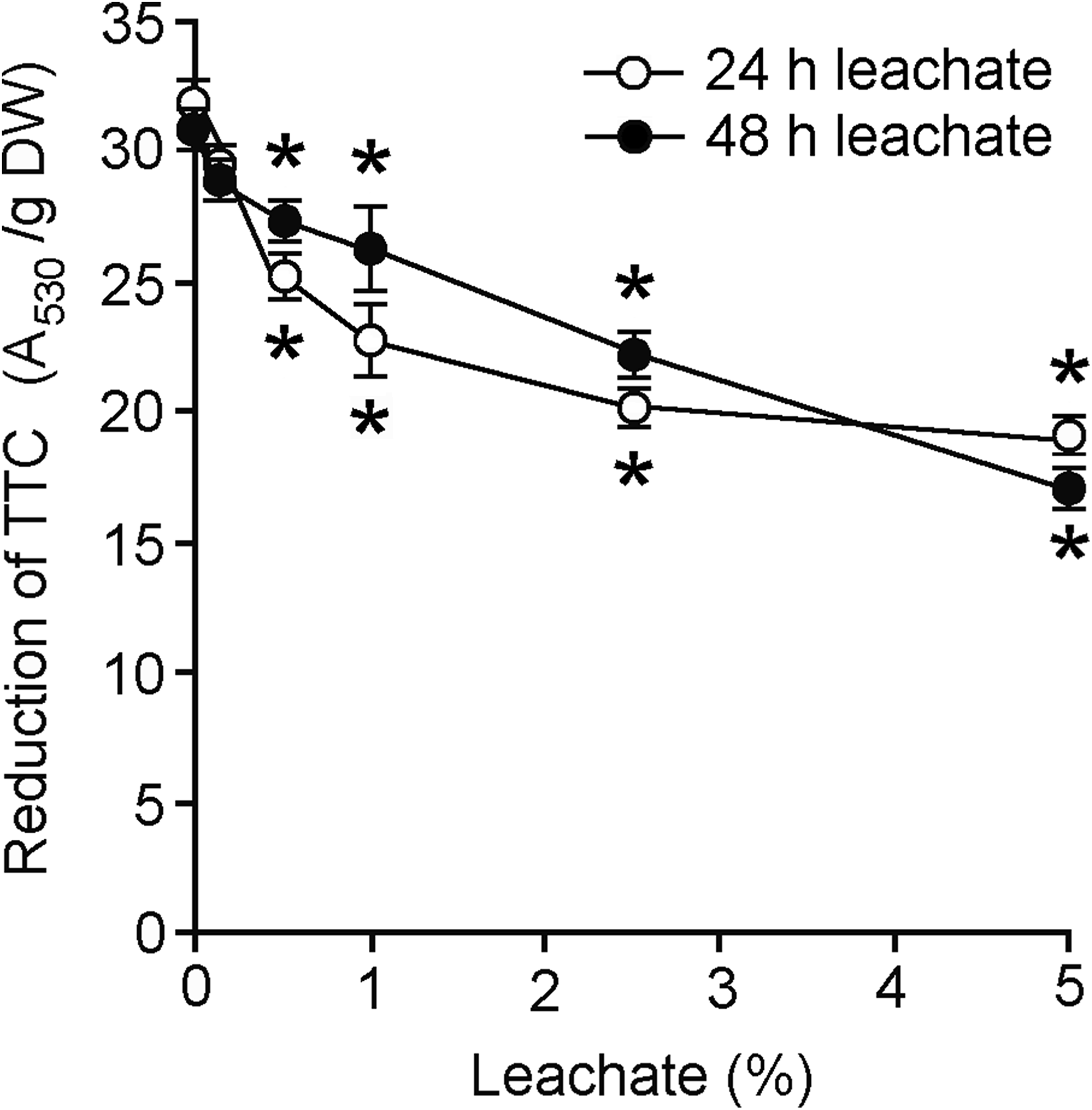

Supplement: Supplementary file 2 — Authors’ original file for figure 2 [file 40529_2012_10_MOESM2_ESM.tif]

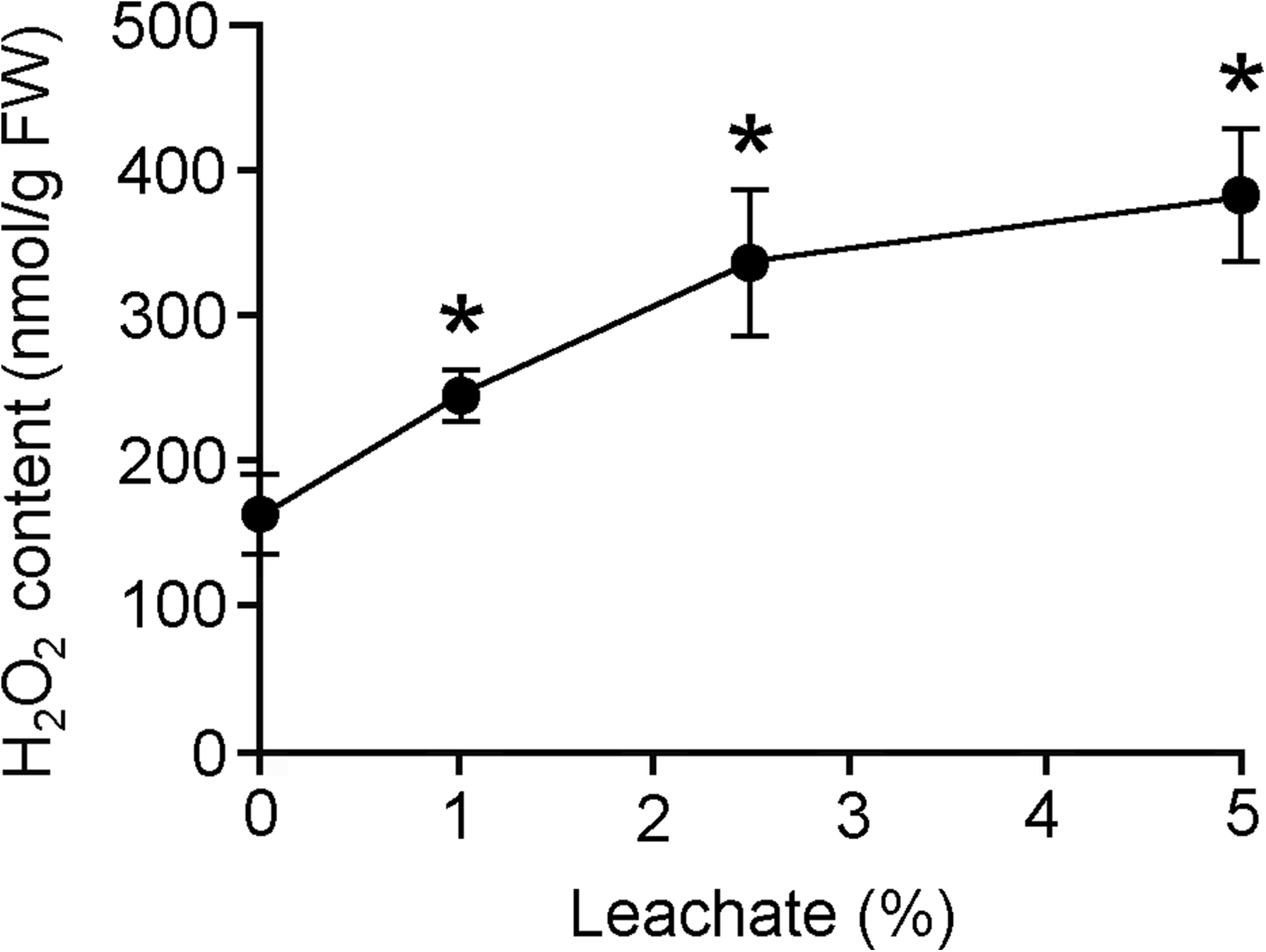

Supplement: Supplementary file 3 — Authors’ original file for figure 3 [file 40529_2012_10_MOESM3_ESM.tif]

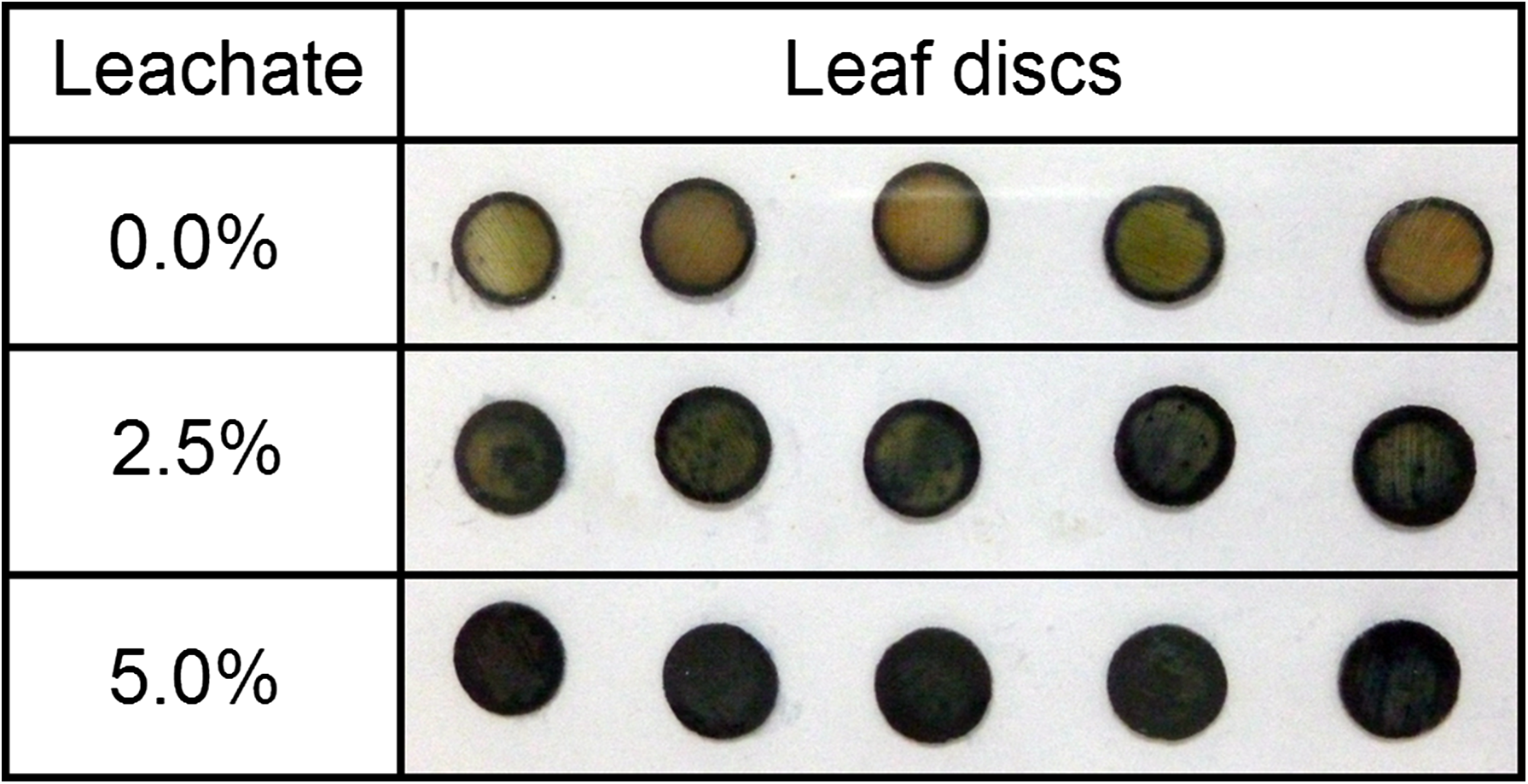

Supplement: Supplementary file 4 — Authors’ original file for figure 4 [file 40529_2012_10_MOESM4_ESM.tif]

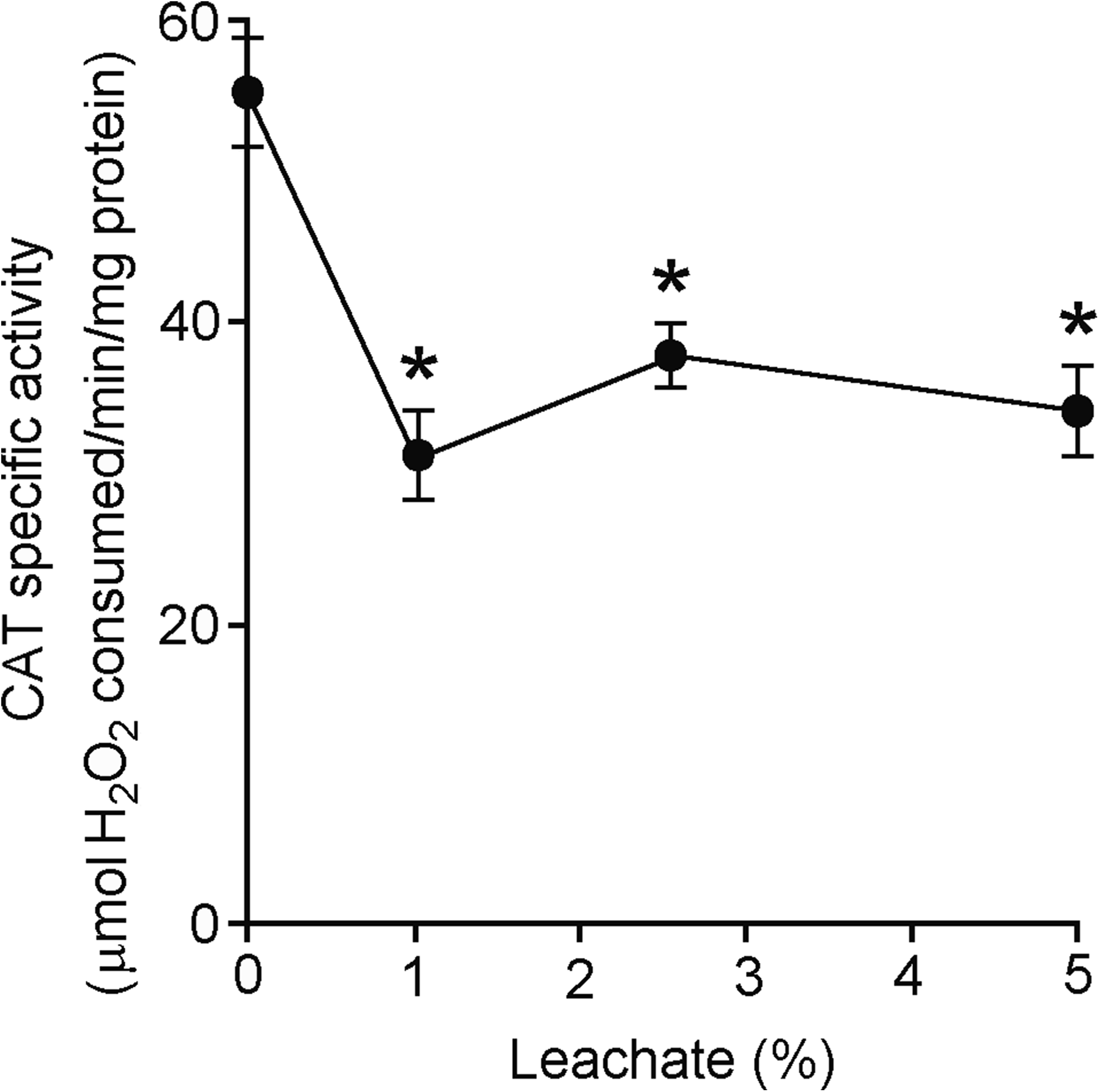

Supplement: Supplementary file 5 — Authors’ original file for figure 5 [file 40529_2012_10_MOESM5_ESM.tif]

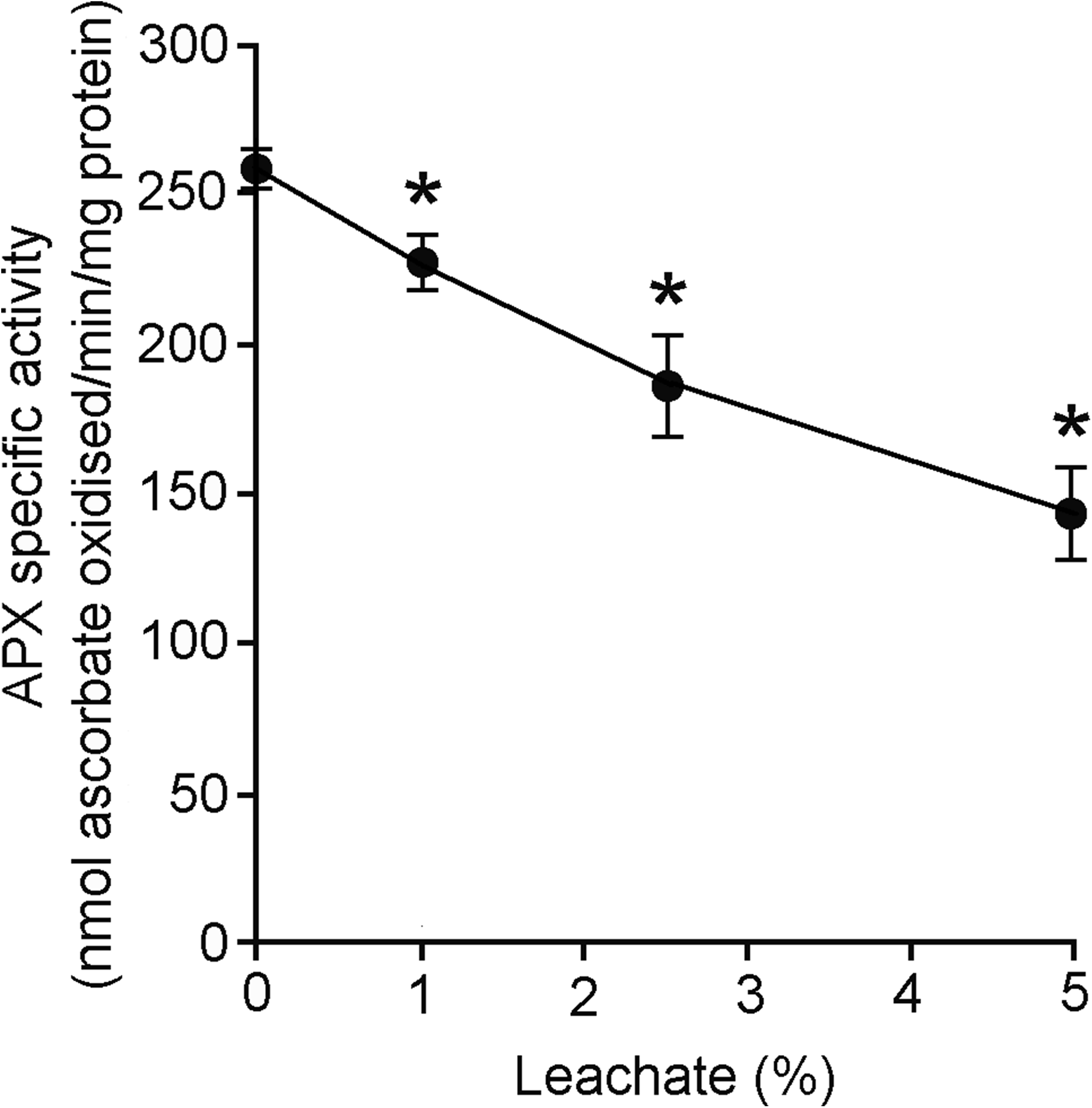

Supplement: Supplementary file 6 — Authors’ original file for figure 6 [file 40529_2012_10_MOESM6_ESM.tif]
